# Health-Risk Behaviors, COVID-19 Preventive Behaviors, and the Impact of the COVID-19 Pandemic on the Working-Age Population of Bangkok, Thailand

**DOI:** 10.3390/ijerph192013394

**Published:** 2022-10-17

**Authors:** Sutham Nanthamongkolchai, Pimsurang Taechaboonsermsak, Kanasap Tawatting, Wanich Suksatan

**Affiliations:** 1 Department of Family Health, Faculty of Public Health, Mahidol University, Bangkok 10400, Thailand; 2 Faculty of Nursing, HRH Princess Chulabhorn College of Medical Science, Chulabhorn Royal Academy, Bangkok 10210, Thailand

**Keywords:** health-risk behaviors, COVID-19 preventive behaviors, impact of COVID-19 pandemic, working-age population, online survey

## Abstract

Although studies have extensively discussed the effects of COVID-19 on global health behaviors, little is known about the extent of COVID-19 preventive behaviors and their negative effects on the working-age population of Bangkok, Thailand. Therefore, this study aimed to examine health-risk behaviors, COVID-19 preventive behaviors, and the impact of the COVID-19 pandemic among the Thai working-age population. An online cross-sectional survey was conducted among working-age adults through a self-administered questionnaire. Data were analyzed with descriptive statistics and stepwise multiple regression. Of the 313 Thai participants (227 females, 72.5%), 84.0% had high levels of health-risk behaviors for preventing COVID-19 infection, most respondents (89.1%) had high levels of knowledge about COVID-19, and most respondents (61.7%) had high levels of overall COVID-19 preventive behaviors. Stepwise multiple regression revealed that health-risk behaviors (β = 0.445), knowledge of COVID-19 (β = 0.148), gender (β = 0.145), and age (β = 0.133) were predictive of COVID-19 preventive behaviors. Additionally, most respondents (48.9%) had moderate overall effects from COVID-19. Based on our findings, these factors must be considered when implementing public policies to improve COVID-19 preventive behaviors among the currently employed working-age population. In addition, appropriate interventions must be established and evaluated for the pandemic’s long-term effects.

## 1. Introduction

Coronavirus disease 2019 (COVID-19), caused by SARS-CoV-2, presents with respiratory symptoms and can be transmitted from person to person through droplets of mucus and saliva expelled when a patient coughs or sneezes [1]. As of 27 September 2022, there had been 612,724,171 people infected worldwide, with 6,517,123 deaths [2]. In Thailand, there is a cumulative total of 4,679,022 confirmed cases, with 32,736 deaths [2]. People around the globe have been affected by COVID-19 (e.g., physical and psychological health problems), causing difficulty to activities of daily life [3].

Several countries, including Thailand, are currently trying to deal with the pandemic through encouraging behaviors such as hand washing, appropriate mask wearing, and social distancing, along with reducing gathering sizes to prevent and control the spread of COVID-19. However, confirmed cases and death rates in Thailand continue to increase, especially in crowded areas (e.g., large cities). Thai people have altered their actions and lifestyle choices to avoid contracting COVID-19 in what has been termed the “new normal”, which may be necessary to stop COVID-19 from emerging again in subsequent outbreaks and causing new COVID-19 pandemic waves [4].

New waves of infection are more common among working-age populations in workplaces, factories, tourist attractions, and entertainment venues [5]. This can lead to the spread of infection among family members and communities. The outbreak of COVID-19 has impacted the economy, society, education, and health [6,7,8]. Prior research conducted during the COVID-19 pandemic in Thailand demonstrated a correlation between public anxiety and knowledge about the virus and COVID-19 preventive behaviors in various populations, such as university students [3,9], health-care workers [10], and older adults [11]. However, knowledge about COVID-19, health-risk behaviors, COVID-19 preventive behaviors, and the effects of COVID-19 is required to prevent the spread of infectious and emerging diseases. In particular, there has been an accumulation of high numbers of COVID-19 mutations (Alpha, Beta, Gamma, Delta, and Omicron variants) with the capacity to evade immunity acquired through vaccination or natural infection, as well as therapies based on antibodies [12]. Thus, it is important to identify the most important health-risk behaviors, knowledge, and preventive behaviors related to COVID-19, which may be diminished if public panic subsides, and ensure public policies promote good COVID-19 preventive behaviors.

Previous studies examined COVID-19 preventive behaviors in Thai populations, such as university students [3,9], adults [13], and older adults [11,14]. However, the COVID-19 preventive behaviors and the impact of the COVID-19 pandemic on the working-age population needs to be examined. In light of the negative economic, social, and health effects of the COVID-19 pandemic, working-age populations may be more susceptible to stressors associated with the pandemic than the general population because they continue to engage in activities in person, travel for work, and engage in social interactions in crowded areas, especially in capital cities such as Bangkok. This suggests that working-age populations are a high-risk group that is more prone to infection than other age groups. As a result, this population has a high risk of passing an infection to family members and others if they have inappropriate knowledge and poor COVID-19 preventive behaviors. Therefore, in this study, we aimed to examine health-risk behaviors, knowledge of COVID-19, COVID-19 preventive behaviors, and the impact of the COVID-19 pandemic among the working-age population in Bangkok, Thailand. We hypothesized that: (a) sociodemographic characteristics, health-risk behaviors, and knowledge of COVID-19 are associated with COVID-19 preventive behaviors and (b) the COVID-19 pandemic effects on the working-age population. The results of our study may serve as the basis for planning and developing guidelines for health promotion among the working-age population to prevent the further spread of COVID-19.

## 2. Materials and Methods

### 2.1. Research Design and Sampling

In this study, a cross-sectional research design was employed in creating an online survey using Google Forms and written in Thai, with medical terms for symptoms written and explained in public language. All questionnaires were sent to participants through social media (primarily Facebook and Line App) for distribution to a working-age population. The formula used by Daniel [10] was employed to determine the sample size for this study, and ultimately, 313 Thai workers were included in this study. Convenience and snowball sampling methods were used to recruit participants, and inclusion criteria were (a) working-age population aged 18–59 years (b) who lived in Bangkok, Thailand, during the COVID-19 outbreak and were (c) able to answer an online questionnaire. The exclusion criteria included anyone who felt uncomfortable, lacked the time, or was not interested in participating for some other reason. The 313 participants, recruited from 20 November 2021, to 10 February 2022, took approximately 30–45 min to complete the questionnaire, and there were no incentives offered to the participants. Adjustments were made such that each participant only submitted one response, although participants occasionally requested the ability to submit multiple responses if they were also completing a questionnaire for relatives who were unable to use the electronic format. Responses were thoroughly examined to ensure there were no duplicates.

#### 2.1.1. Health-Risk Behaviors Related to COVID-19 Infection

The questionnaire measuring health-risk behaviors regarding COVID-19 infection was developed by the research team and consisted of 10 items answered on a 4-point scale, with 4 = “very” and 1 = “not at all”. The total score ranged from 10 to 40. According to Bloom [15], mean scores were divided into three levels: low level (1–59), moderate level (60–79), and high level (80–100), with higher scores indicating greater perceived COVID-19 risk. The questionnaire had been tested and its validity and reliability confirmed, with a content validity index (CVI) of 0.87. The Cronbach’s alpha coefficient of this questionnaire was 0.85.

#### 2.1.2. Knowledge of COVID-19

The questionnaire measuring knowledge about COVID-19 was a 15-item scale developed by Upake et al. [11] and validated in a Thai context. This questionnaire consisted of four subdimensions: (1) disease cause, (2) signs and symptoms, (3) incubation period, and (4) transmission route. The items were rated on a scale of 1 to 0, with 1 representing a correct answer and 0 for an incorrect answer; the total score ranged from 0 to 15. According to Bloom [15], the mean scores are divided into three levels: low (15–59), moderate (60–79), and high (80–100), with a higher score indicating better knowledge about COVID-19. In this study, the Kuder–Richardson method was used to analyze internal consistency reliability, with a score of 0.79.

#### 2.1.3. COVID-19 Preventive Behaviors

The COVID-19 preventive behaviors questionnaire was developed Upake et al. [11] and validated in a Thai context. This questionnaire was a 15-item scale with three subdimensions: (1) strength-building behaviors, (2) compliance with DMHTT measures (D = distancing; M = mask wearing; H = handwashing; T = testing, including temperature taking and getting tested for COVID; and T = Thai Cha Na application check, which is a user registration system used for visiting certain areas, places, or buildings at risk of COVID-19 infection and access to personal travel information); and (3) screening and vaccinations. Each item was scored on a 5-point scale, where 1 = not practiced and 5 = practiced often, with a total score of 15 to 75. According to Bloom [15], the mean scores were divided into three levels: low (15–59), moderate (60–79), and high (80–100), where a higher score suggests more COVID-19 preventive behaviors. In this study, Cronbach’s alpha for the COVID-19 preventive behaviors questionnaire was 0.80.

#### 2.1.4. Effects of COVID-19

The questionnaire on the effects of COVID-19 was developed by Kasatpibal et al. [16], and validated in a Thai context. This questionnaire was a 16-item scale consisting of four subdimensions: (1) effects of health and disease prevention; (2) family effects; (3) economic effects; and (4) psychological components. Each item was scored on a 5-point scale where 1 = “strongly disagree” and 5 = “strongly agree”, with a total score ranging from 16 to 80 and mean scores divided into three levels [15]: low (16–59), moderate (60–79), and high (80–100). In this regard, a higher score represented more effects from COVID-19. This questionnaire was tested, and its validity and reliability confirmed, with a CVI and Cronbach’s alpha coefficient of 0.93 and 0.84, respectively.

#### 2.1.5. Sociodemographic Information

The sociodemographic information encompassed the six variables of age, sex, educational level, marital status, monthly income, and number of family members.

### 2.2. Data Analyses

The sociodemographic information was analyzed with descriptive statistics by displaying frequency, percentages, mean scores, and standard deviation. We used Pearson’s product moment correlation coefficient to determine the relationships between participants’ characteristics, health-risk behaviors, and knowledge about COVID-19. In addition, predicting the preventive behaviors regarding COVID-19 was analyzed using stepwise multiple regression analysis, and assumptions of multivariate normality were met. The significance level was set at *p* < 0.05 for all analyses. All statistical analyses were performed using IBM SPSS 25.0 software (IBM Corp., Armonk, NY, USA).

## 3. Results

### 3.1. Characteristics of Participants

Of the 313 participants in the study, 72.5% (*n* = 227) were female. The average age was 34.6 years (SD ± 10.23), and 136 (43.5%) were aged 19–30 years. A total of 84.7% (*n* = 256) held bachelor’s degrees or had higher education levels. Most of the participants (71.9%; *n* = 225) were currently single, and the average income was USD 734.08 (SD ± 172.42), which was generally uniformly spread between USD 57.14 and 1000 per month (74.7%; *n* = 234). The average family size was 3.2 persons (SD ± 1.24), and most participants (41.6%; *n* = 130) lived with 3–4 family members, while 37.7 % (*n* = 128) lived with 1–2 family members (see Table 1).

### 3.2. Participant Levels of Health-Risk Behaviors and Knowledge about COVID-19

**Figure 1:**
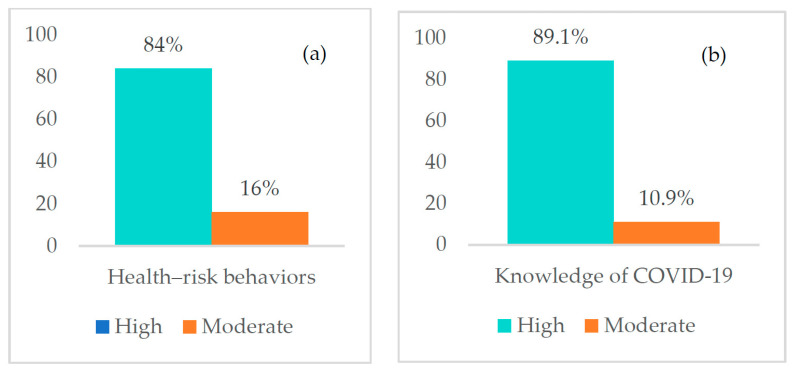
Participant levels of (**a**) health-risk behavior levels and (**b**) knowledge about COVID-19.

Of the 313 participants in the study, 84.0% (*n* = 263) had high levels of health-risk behaviors regarding COVID-19 infection (see Figure 1a). In addition, most participants (89.1%, *n* = 279) had a high level of knowledge (see Figure 1b).

### 3.3. COVID-19 Preventive Behaviors Levels

**Figure 2:**
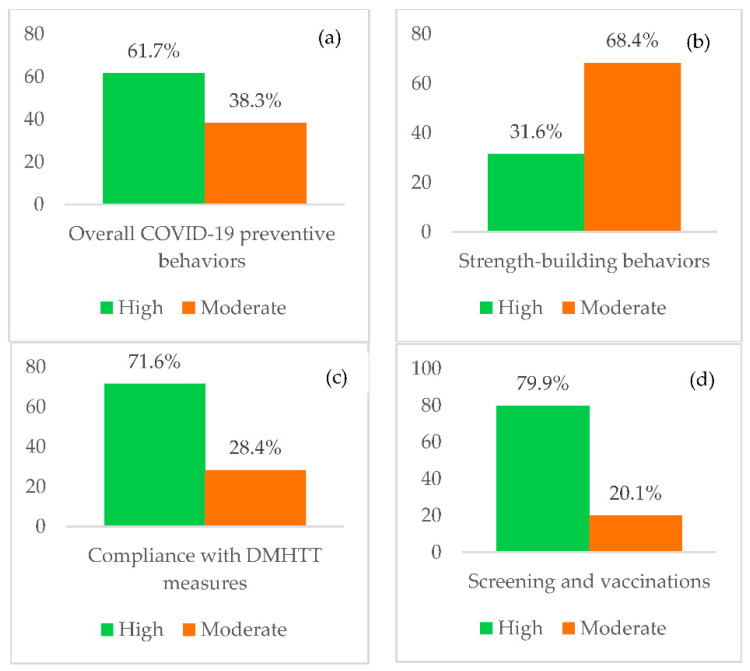
Participant levels of (**a**) overall COVID-19 preventive behaviors; (**b**) strength-building behaviors; (**c**) compliance with DMHTT measures; and (**d**) screening and vaccinations.

According to the findings, most respondents had high overall levels of COVID-19 preventive behaviors (61.7%) (Figure 2a). When preventive behaviors were considered in each subdimension, most respondents were found to have strength-building behaviors at moderate levels (68.4%), followed by high levels (31.6%) (Figure 2b). In terms of DMHTT measures, most of the respondents had high levels of compliance (71.6%) (Figure 2c). Additionally, most of the respondents had high levels of compliance with screening and vaccinations (79.9%) (Figure 2d).

### 3.4. Effects of COVID-19 among Study Participants

According to our findings, most of the participants had moderate overall effects from COVID-19 (48.9%), followed by high levels of effects (32.6%) and low levels (18.5%). When the effects of COVID-19 were considered in separate areas, much of the working-age population was found to have health and disease prevention effects at moderate levels (47.3%), followed by high (36.7%) and low (16.0%) levels. In the area of family effects, most participants had family effects at high levels (41.2%), followed by moderate (32.9%) and low (25.9%) levels. Most participants had high levels of economic effects (44.4%) and high levels of psychological effects (52.1%) (Table 2).

### 3.5. Factors Correlated with Level of COVID-19 Preventive Behaviors

As shown in Table 3, our results showed that age (r = 0.226), health-risk behaviors (r = 0.475), and knowledge about COVID-19 (r = 0.116) were associated with COVID-19 preventive behaviors (*p* < 0.05), while income and number of family members were uncorrelated (*p* > 0.05).

### 3.6. Factors Influencing COVID-19 Preventive Behaviors

In this study, the prediction of preventive behaviors of COVID-19 was measured with stepwise multiple regression analysis. We found that health-risk behaviors (β = 0.445), knowledge about COVID-19 (β = 0.148), gender (β = 0.145), and age (β = 0.133) were predictive of COVID-19 preventive behaviors among the working-age population, accounting for approximately 28.1% of the variance (Table 4).

## 4. Discussion

This study provides crucial information regarding the health-risk behaviors, COVID-19 preventive behaviors, and impacts of the COVID-19 pandemic among a working-age population in Bangkok, Thailand. We found that the participating working adults had a high level of self-assessment of health-risk behaviors for COVID-19 infection, at 84.0%. It can be explained that the high risk of contracting COVID-19 may be prevented by having good habits of masking regularly, washing hands often, and keeping social distances from others [14,17].

Most of the respondents had high levels of overall COVID-19 preventive behaviors (61.7%). This is likely due to Thailand implementing measures to prevent COVID-19, and thus, most working-age people exercising appropriate COVID-19 preventive behaviors [18]. Moreover, working-age people are accustomed to activities or working outside the home amid a crisis, resulting in this group of people practicing good self-care behaviors in their daily lives [3]. Additionally, the emphasis on the severity of the disease and the impact of COVID-19 infection on their health and life encouraged working adults to adopt appropriate disease prevention behaviors to enable them to continue working [19,20]. Our results also showed that most respondents had a high level of compliance with the DMHTT measures, suggesting that strict adherence to government policies and disease prevention practices is accepted as part of daily life [21,22,23].

This study revealed that health-risk behaviors, knowledge of COVID-19, gender, and age were associated with COVID-19 preventive behaviors among Thai workers. We also found that predictors of COVID-19 preventive behaviors were health-risk behaviors, knowledge about COVID-19, gender, and age. Health-risk behaviors regarding COVID-19 infection had the greatest impact on the COVID-19 preventive behaviors of study respondents. These results are essential for developing effective guidelines to battle COVID-19 [24,25]. Consistent with prior research, our findings indicate that perceived risk and barriers to preventing COVID-19 infection are the most influential factors in behaviors that can prevent COVID-19 infection in the workplace [26].

Knowledge about COVID-19 also influenced COVID-19 preventive behaviors among the people of working age in this study. We discovered that the working-age population had high levels of knowledge about COVID-19, suggesting that knowledge about COVID-19, particularly among working-age Thais, is crucial for combating the COVID-19 pandemic [4,11,27]. Consistent with prior research, our findings indicated a positive correlation between COVID-19 knowledge and COVID-19 preventive behaviors [28,29].

In our study, gender was one factor that influenced COVID-19 preventive behaviors among working-age people, with our findings revealing that females had high levels of COVID-19 preventive behaviors. The findings of our study are in line with those of prior studies showing that male and female study participants engaged in significantly different psychosocial factors related to COVID-19 preventive behaviors [30,31]. According to Pender’s Health Promotion Model (HBM), age is one of the personal factors that can be relevant to an individual’s behaviors and are correlated with health status [32]. This is also in line with prior research from Iran and Israel [33,34], in which it was discovered that most female respondents demonstrated COVID-19 preventive behaviors at higher levels than males.

Age was the last factor that influenced behaviors for preventing COVID-19. Older workers in our study had high levels of COVID-19 preventive behaviors, which may be explained by older workers adopting good behaviors to prevent COVID-19 through compliance with DMHTT measures and screening and vaccinations, along with strength-building behaviors [14]. While all age groups are susceptible to COVID-19 infection, older adults are more likely to experience severe symptoms due to infection [35,36]. Consistent with Pender’s HBM, age is one of the personal factors that can affect an individual’s behaviors and be related to health status [32]. Previous studies have revealed that middle-aged and older persons were more likely to see their own and each other’s health practices as adequate for minimizing COVID-19 transmission, whereas younger adults were more likely to consider their own and each other’s health behaviors as inadequate [37,38].

Additionally, this study found that 48.9% of respondents had high levels of overall effects from the COVID-19 pandemic. The participants were most affected at high levels in psychological aspects (52.1%), followed by economic and family effects (44.4% and 41.2%, respectively). Previous studies also revealed that the COVID-19 pandemic economic, psychological, and family relationship effects [6,7,8]. Such impacts likely caused working-age groups to adjust to daily life, work, and taking care of themselves and their families, leading to better behaviors for preventing COVID-19 infection. However, we found that the effects of COVID-19 did not influence the knowledge or the adherence to preventive on these participants. As such, the results of this study are unclear at this stage, and further studies are required.

Despite the importance of these findings for a better understanding of health-risk behaviors associated with COVID-19 infection and the impact of the COVID-19 pandemic on preventive behaviors among Thai working-age populations, we acknowledge that there are significant limitations. First, the sample size of this study was small, possibly due to issues with accessibility to the internet or electronic devices. Further research is needed with large sample sizes and access to online questionnaires without restrictions. Second, the Google Form questionnaire (online survey) was sent to participants through social media (Facebook and Line App); thus, social desirability and potentially biased ratings of self-assessed behavior cannot be ruled out, which may have led to bias and skewing of results. Third, the findings of our study may not be generalizable to the working-age populations in Bangkok because our study was not based on random sampling. Additionally, convenience and snowball sampling were used in this study, and all of the participants voluntarily participated, which could indicate selection bias. Finally, we did not assess the negative effects of environmental factors or climate change on other factors related to COVID-19 preventive behaviors among Thai working-age respondents. Future studies are needed to determine the long-term impact of the pandemic.

## 5. Conclusions

The present study identified health-risk behaviors of COVID-19 infection and knowledge about COVID-19 that were associated with COVID-19 preventive behaviors. The findings also revealed that the predictors of COVID-19 preventive behaviors among working-age respondents were health-risk behaviors, knowledge about COVID-19, gender, and age. In addition, most participants had high levels of effects from the COVID-19 pandemic in psychological and economic aspects. Based on our results, these factors would be useful for healthcare providers and policymakers to consider when implementing proper interventions for a better understanding of how to improve COVID-19 preventive behaviors among working-age populations. Future studies (e.g., longitudinal study, in-depth interviews) need to be established and evaluated for the long-term impact of the pandemic.

## Figures and Tables

**Table 1:** Participants’ demographic data.

Factors	Number	Percentage
Sex		
Female	227	72.5
Male	86	27.5
Age (years)		
18–30	136	43.5
31–40	87	27.8
41–50	63	20.1
51–59	27	8.6
(mean = 34.6, SD ± 10.23)		
Educational level		
High school or lower	48	15.3
Bachelor’s degrees or higher	265	84.7
Marital status		
Single	230	73.5
Married	83	26.5
Income (USD)		
57–429	94	30.0
430–714	83	26.5
715–1000	57	18.2
>1000	79	24.8
(mean = 734.08, SD ± 172.42)		
Family size (person)		
1–2	118	37.70
3–4	130	41.53
>5	65	20.77
(mean = 3.2, SD ± 1.24)		

**Table 2:** Characteristics of effects from COVID-19.

	Number	Percentage
Overall effects from COVID-19		
Low	58	18.5
Moderate	153	48.9
High	102	32.6
Health and disease prevention		
Low	50	16.0
Moderate	148	47.3
High	115	36.7
Family		
Low	81	25.9
Moderate	103	32.9
High	129	41.2
Economic		
Low	85	27.2
Moderate	89	28.4
High	139	44.4
Psychological		
Low	52	16.6
Moderate	98	31.3
High	163	52.1

**Table 3:** Factors correlated with COVID-19 preventive behaviors.

Variables	COVID-19 Preventive Behaviors	
	Coefficient Correlation (r)	*p*-Value
Age	0.226	<0.001
Income	−0.034	0.55
Number of family member	0.102	0.07
Health-risk behaviors	0.475	<0.001
Knowledge of COVID-19	0.116	0.040

**Table 4:** Variables predicting participant’s COVID-19 preventive behaviors.

Factors	B	Beta	T	*p*-Value
Health-risk behaviors	0.798	0.445	8.929	<0.001
Knowledge of COVID-19	0.660	0.148	3.050	0.002
Gender (female vs. male *)	2.130	0.145	3.005	0.003
Age	0.086	0.133	2.663	0.008

Notes: R^2^ = 0.281, * Reference group

## Data Availability

Not applicable.
